# Ethyl 6-amino-5-cyano-4-phenyl-2,4-di­hydro­pyrano[2,3-*c*]pyrazole-3-carboxyl­ate dimethyl sulfoxide monosolvate

**DOI:** 10.1107/S1600536814013270

**Published:** 2014-06-18

**Authors:** Naresh Sharma, Goutam Brahmachari, Bubun Banerjee, Rajni Kant, Vivek K. Gupta

**Affiliations:** a Post-Graduate Department of Physics & Electronics, University of Jammu, Jammu Tawi 180 006, India; b Laboratory of Natural Products & Organic Synthesis, Department of Chemistry, Visva-Bharati University, Santiniketan 731 235, West Bengal, India

## Abstract

In the asymmetric unit of the title compound, C_16_H_14_N_4_O_3_·C_2_H_6_OS, there are two independent main mol­ecules (*A* and *B*) and two dimethyl sulfoxide solvent mol­ecules. In mol­ecule *A*, the pyran ring is in a flattened sofa conformation, with the *sp* ^3^-hydridized C atom forming the flap. In mol­ecule *B*, the pyran ring is in a flattened boat conformation, with the *sp* ^3^-hydridized C atom and the O atom deviating by 0.073 (3) and 0.055 (3) Å, respectively, from the plane of the other four atoms. The mean planes the pyrazole and phenyl rings form dihedral angles of 84.4 (2) and 84.9 (2)°, respectively, for mol­ecules *A* and *B*. In the crystal, N—H⋯O and N—H⋯N hydrogen bonds link the components of the structure into chains along [010]. In both solvent mol­ecules, the S atoms are disordered over two sites, with occupancy ratios of 0.679 (4):0.321 (4) and 0.546 (6):0.454 (6).

## Related literature   

For background to the biological activity of synthetic pyrano[2,3-*c*] pyrazole compounds, see: Nasr *et al.* (2002 ▶); Ismail *et al.* (2003 ▶); Foloppe *et al.* (2006 ▶); Mohamed *et al.* (2010 ▶); Zonouz *et al.* (2012 ▶); Kuo *et al.* (1984 ▶); Zaki *et al.* (2006 ▶); Ahluwalia *et al.* (1997 ▶); Bhavanarushi *et al.* (2013 ▶). For the synthesis of the title compound, see: Brahmachari & Banerjee (2014 ▶). For a related structure, see: Topno *et al.* (2011 ▶). For standard bond-length data, see: Allen *et al.* (1987 ▶). For ring conformations, see: Duax & Norton (1975 ▶).
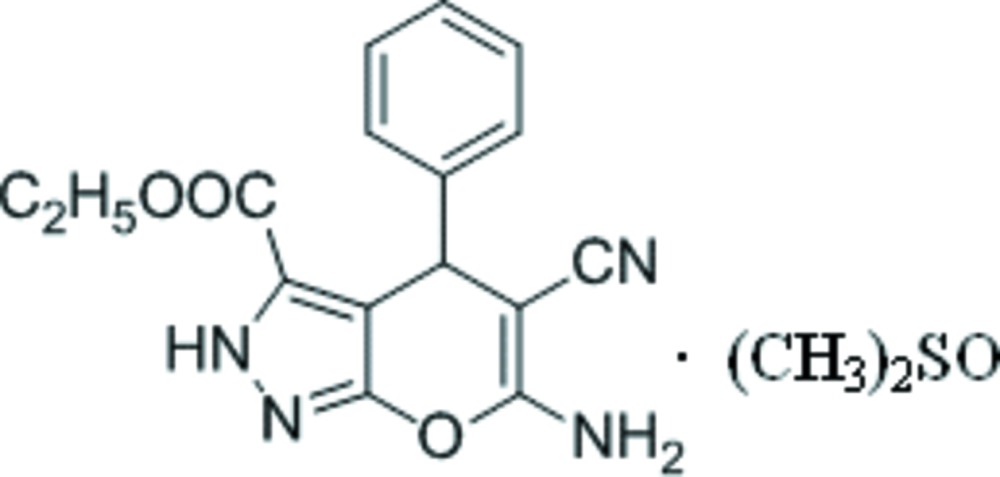


## Experimental   

### 

#### Crystal data  

C_16_H_14_N_4_O_3_·C_2_H_6_OS
*M* *_r_* = 388.44
Monoclinic, 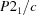

*a* = 28.018 (5) Å
*b* = 9.196 (5) Å
*c* = 15.396 (5) Å
β = 93.376 (5)°
*V* = 3960 (3) Å^3^
*Z* = 8
Mo *K*α radiation
μ = 0.19 mm^−1^
*T* = 293 K
0.30 × 0.20 × 0.20 mm

#### Data collection  

Oxford Diffraction Xcalibur Sapphire3 diffractometer
Absorption correction: multi-scan (*CrysAlis RED*; Oxford Diffraction, 2010 ▶) *T*_min_ = 0.805, *T*_max_ = 1.000
13653 measured reflections
6946 independent reflections
3460 reflections with *I* > 2σ(*I*)
*R*_int_ = 0.052

#### Refinement  

*R*[*F* ^2^ > 2σ(*F* ^2^)] = 0.068
*wR*(*F* ^2^) = 0.210
*S* = 1.00
6946 reflections
510 parameters
H-atom parameters constrained
Δρ_max_ = 0.32 e Å^−3^
Δρ_min_ = −0.24 e Å^−3^

### 

Data collection: *CrysAlis PRO* (Oxford Diffraction, 2010 ▶); cell refinement: *CrysAlis PRO*; data reduction: *CrysAlis PRO*; program(s) used to solve structure: *SHELXS97* (Sheldrick, 2008 ▶); program(s) used to refine structure: *SHELXL97* (Sheldrick, 2008 ▶); molecular graphics: *ORTEP-3 for Windows* (Farrugia, 2012 ▶); software used to prepare material for publication: *PLATON* (Spek, 2009 ▶).

## Supplementary Material

Crystal structure: contains datablock(s) I, New_Global_Publ_Block. DOI: 10.1107/S1600536814013270/lh5712sup1.cif

Structure factors: contains datablock(s) I. DOI: 10.1107/S1600536814013270/lh5712Isup2.hkl

Supporting information file. DOI: 10.1107/S1600536814013270/lh5712Isup3.cml

CCDC reference: 1006444

Additional supporting information:  crystallographic information; 3D view; checkCIF report

## Figures and Tables

**Table 1:** Hydrogen-bond geometry (Å, °) *Cg* is the centroid of the N1*B*–C9*B*/C8*B* ring.

*D*—H⋯*A*	*D*—H	H⋯*A*	*D*⋯*A*	*D*—H⋯*A*
N2*A*—H2*A*⋯O1*S*	0.86	1.90	2.737 (4)	165
N2*B*—H2*B*⋯O2*S* ^i^	0.86	1.90	2.750 (5)	168
N12*A*—H50*A*⋯N11*B* ^ii^	0.86	2.19	3.024 (5)	164
N12*A*—H40*A*⋯O13*A* ^iii^	0.86	2.11	2.958 (4)	170
N12*B*—H50*B*⋯N11*A* ^iv^	0.86	2.23	3.072 (5)	165
N12*B*—H40*B*⋯O13*B* ^v^	0.86	2.10	2.945 (4)	168

Symmetry codes: (i) 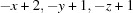; (ii) 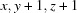; (iii) 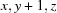; (iv) 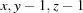; (v) 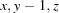.

**Fig. 1.**
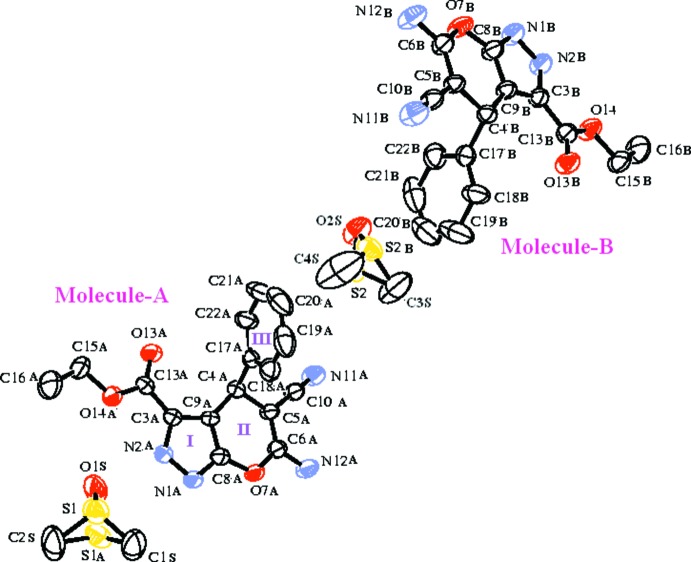
The asymmetric unit of (I). with ellipsoids drawn at the 40% probability level. H atoms are shown as small spheres of arbitrary radii.

**Fig. 2.**
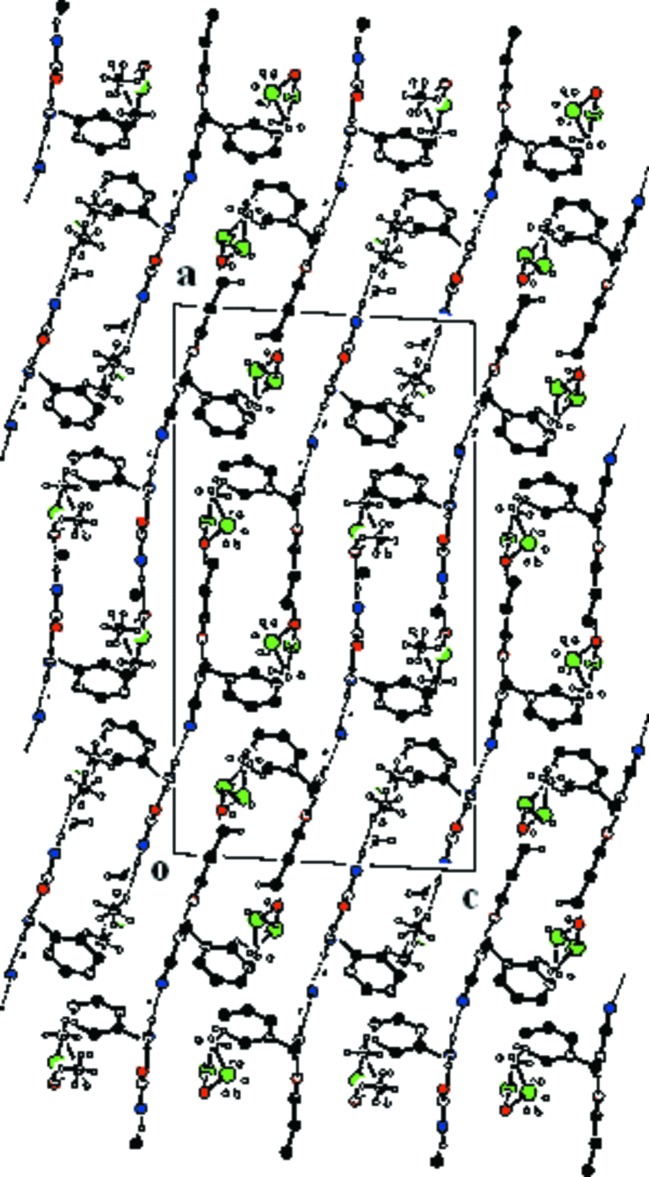
Part of the crystal structure with hydrogen bonds shown as dahed lines.

## supplementary crystallographic information

### S1. Comment

Pyrano[2,3-*c*]pyrazole scaffolds represent a "privileged" structural motif well distributed in naturally occurring compounds with a broad spectrum of significant biological activities. Recently, a series of synthetic pyrano[2,3-*c*] pyrazole compounds have been evaluated and possess potent bactericidal (Nasr *et al.*, 2002), insecticidal (Ismail *et al.*, 2003), molluscicidal (Zonouz *et al.*, 2012), analgestic (Kuo *et al.*, 1984), anti-inflammatory activities (Zaki *et al.*, 2006), hypotensive (Ahluwalia *et al.*, 1997), hypoglycemic, and anticancer agents (Mohamed *et al.*, 2010; Bhavanarushi *et al.*, 2013). They are also potential inhibitors of human Chk1 kinase (Foloppe *et al.*, 2006). Hence, investigation of the structural features of biologically relevant pyrano[2,3-*c*]pyrazole derivatives is of both scientific and practical interest. In continuation of our efforts to develop useful synthetic protocols for biologically significant molecules, we report herein an efficient and environmentally benign synthesis and the crystal structure of the title compound (I)

The asymmetric unit of the title compound is shown in Fig. 1. There are two crystallographically independent molecules (A and B). The geometry of both molecules is similar and in terms of their bond lengths and bond angles and are in good agreement with the standard values (Allen *et al.*, 1987) and corresponds to those observed in a related structure (Topno *et al.*, 2011). The double bond length C13A═O13A [1.212 (5) Å] and C13B═O13B [1.210 (5) Å] indicates the C═O double bond slightly longer than that observed for carbonyl bonds, probably because atoms O13A and O13B are involved in intermolecular N—H···O hydrogen bonds. The pyran ring (ring II) adopts a flattned sofa conformation in molecule A and a flattened boat conformation in molecule B with asymmetry parameters [ΔCs(C4) = 2.73] (molecule A) and [ΔCs(C4)) = 1.03, ΔC2(C4—C5) = 1.14] (molecule B) (Duax & Norton, 1975). The mean planes the pyrazole (ring I) and phenyl (ring III) rings from dihedral angles of 84.4 (2)° and 84.9 (2)°, respectively for molecules A and B. In the crystal, N—H···O and N—H···N hydrogen bonds link the components of the structure into chains along [010] (Fig. 2). In both solvent molecules, the S atoms are disordered over two sites with occupancy ratios of 0.679 (4):0.321 (4) and 0.546 (6):0.454 (6).

### S2. Experimental

The synthesis of the title compound, ethyl 6-amino-5-cyano-4-phenyl-2, 4-dihydropyrano[2,3-*c*]pyrazole-3-carboxylate (I), was carried out *via* one-pot multi-component reaction in aqueous ethanol using low-cost and environmentally benign urea as catalyst at room temperature. An oven-dried screw cap test tube was charged with a magnetic stir bar, diethyl acetylenedicarboxylate (0.170 g, 1.0 mmol) and hydrazine hydrate (0.050 g, 1 mmol); the reaction mixture was then stirred at room temperature for about 10 min. After that, benzaldehyde (0.106 g, 1 mmol), malononitrile (0.066 g, 1.1 mmol), urea (0.007 g, 10 mol % as organo-catalyst) and EtOH:H_2_O (1:1 *v*/*v*; 4 ml) was added in a sequential manner (Brahmachari and Banerjee, 2014). The reaction mixture was then stirred vigorously at room temperature and the stirring was continued for 10 h. The progress of the reaction was monitored by TLC. On completion of the reaction, a solid mass was precipitated out, filtered off and repeatedly washed with aqueous ethanol to obtain a crude product which was purified just by recrystallization from ethanol without carrying out column chromatography. The structure of (I) was confirmed by analytical as well as spectral studies including ^1^H NMR, ^13^C NMR, and TOF-MS. The single crystal was obtained from DMSO as a solvent. For crystallization 50 mg of (I) dissolved in 5 ml DMSO was left for several days at ambient temperature which yielded white block shaped crystals. Ethyl 6-amino-5-cyano-4-phenyl-2,4-dihydropyrano[2,3-*c*] pyrazole-3-carboxylate (1). White solid. Yield 91%. Mp: 521–523 K. ^1^H NMR (400 MHz, DMSO-d_6_) δ /p.p.m.: 13.76 (1*H*, s, NH), 7.28 (2*H*, t, *J* = 7.2 Hz, aromatic H), 7.19 (1*H*, t, *J* = 7.2 Hz, aromatic H), 7.10 (2*H*, d, *J* = 7.2 Hz, aromatic H), 7.03 (2*H*, s, NH_2_), 4.76 (1*H*, s, CH), 4.07 (2*H*, q, *J* = 7.2 \ 6.8 Hz, CH_3_), 1.03 (3*H*, t, *J* = 7.2 & 6.8 Hz, CH_3_). ^13^C NMR (100 MHz, DMSO-d_6_) δ /p.p.m.: 160.43, 158.56, 156.01, 145.31, 129.48, 128.66 (2 C), 127.73 (2 C), 127.04, 120.71, 104.03, 61.24, 58.32, 37.38, 14.14. TOF-MS: 333.0961 [*M*+Na]^+^. Elemental analysis: Calcd. (%) for C_16_H_14_N_4_O_3_: C, 61.93; H, 4.55; N, 18.06; found: C, C, 61.96; H, 4.53; N, 18.04.

### S3. Refinement

All H atoms were geometrically fixed and allowed to ride on their parent C atoms, with C—H distances of 0.93–0.98 Å, N—H = 0.86Å and with *U*_iso_(H) = 1.2*U*_eq_(C,N).

### Crystal data

**Table:**

C_16_H_14_N_4_O_3_·C_2_H_6_OS	*F*(000) = 1632
*M**_r_* = 388.44	*D*_x_ = 1.303 Mg m^−^^3^
Monoclinic, *P*2_1_/*c*	Mo *K*α radiation, λ = 0.71073 Å
Hall symbol: -P 2ybc	Cell parameters from 1789 reflections
*a* = 28.018 (5) Å	θ = 4.1–26.7°
*b* = 9.196 (5) Å	µ = 0.19 mm^−^^1^
*c* = 15.396 (5) Å	*T* = 293 K
β = 93.376 (5)°	Block, white
*V* = 3960 (3) Å^3^	0.30 × 0.20 × 0.20 mm
*Z* = 8	

### Data collection

**Table:**

Oxford Diffraction Xcalibur Sapphire3 diffractometer	6946 independent reflections
Radiation source: fine-focus sealed tube	3460 reflections with *I* > 2σ(*I*)
Graphite monochromator	*R*_int_ = 0.052
Detector resolution: 16.1049 pixels mm^-1^	θ_max_ = 25.0°, θ_min_ = 3.4°
ω scans	*h* = −31→33
Absorption correction: multi-scan (*CrysAlis RED*; Oxford Diffraction, 2010)	*k* = −6→10
*T*_min_ = 0.805, *T*_max_ = 1.000	*l* = −18→9
13653 measured reflections	

### Refinement

**Table:**

Refinement on *F*^2^	Primary atom site location: structure-invariant direct methods
Least-squares matrix: full	Secondary atom site location: difference Fourier map
*R*[*F*^2^ > 2σ(*F*^2^)] = 0.068	Hydrogen site location: inferred from neighbouring sites
*wR*(*F*^2^) = 0.210	H-atom parameters constrained
*S* = 1.00	*w* = 1/[σ^2^(*F*_o_^2^) + (0.0876*P*)^2^] where *P* = (*F*_o_^2^ + 2*F*_c_^2^)/3
6946 reflections	(Δ/σ)_max_ < 0.001
510 parameters	Δρ_max_ = 0.32 e Å^−^^3^
0 restraints	Δρ_min_ = −0.24 e Å^−^^3^

### Special details

**Table:**

Experimental. *CrysAlis PRO*, Agilent Technologies, Version 1.171.36.28 (release 01–02-2013 CrysAlis171. NET) (compiled Feb 1 2013,16:14:44) Empirical absorption correction using spherical harmonics, implemented in SCALE3 ABSPACK scaling algorithm.
Geometry. All e.s.d.'s (except the e.s.d. in the dihedral angle between two l.s. planes) are estimated using the full covariance matrix. The cell e.s.d.'s are taken into account individually in the estimation of e.s.d.'s in distances, angles and torsion angles; correlations between e.s.d.'s in cell parameters are only used when they are defined by crystal symmetry. An approximate (isotropic) treatment of cell e.s.d.'s is used for estimating e.s.d.'s involving l.s. planes.
Refinement. Refinement of *F*^2^ against ALL reflections. The weighted *R*-factor *wR* and goodness of fit *S* are based on *F*^2^, conventional *R*-factors *R* are based on *F*, with *F* set to zero for negative *F*^2^. The threshold expression of *F*^2^ > σ(*F*^2^) is used only for calculating *R*-factors(gt) *etc*. and is not relevant to the choice of reflections for refinement. *R*-factors based on *F*^2^ are statistically about twice as large as those based on *F*, and *R*- factors based on ALL data will be even larger.

### Fractional atomic coordinates and isotropic or equivalent isotropic displacement parameters (Å^2^)

**Table:**

	*x*	*y*	*z*	*U*_iso_*/*U*_eq_	Occ. (<1)
N1A	0.53193 (10)	0.7819 (3)	0.8897 (2)	0.0695 (9)	
N2A	0.52515 (10)	0.6372 (3)	0.8921 (2)	0.0677 (9)	
H2A	0.4974	0.5974	0.8917	0.081*	
C3A	0.56660 (11)	0.5602 (3)	0.8952 (2)	0.0561 (9)	
C4A	0.65667 (11)	0.6520 (3)	0.8983 (2)	0.0512 (8)	
H4A	0.6677	0.5907	0.9476	0.061*	
C5A	0.67549 (11)	0.8066 (3)	0.9139 (2)	0.0502 (8)	
C6A	0.64854 (12)	0.9290 (3)	0.9073 (2)	0.0571 (9)	
O7A	0.60010 (8)	0.9282 (2)	0.89259 (17)	0.0674 (7)	
C8A	0.57909 (12)	0.7925 (3)	0.8921 (3)	0.0616 (9)	
C9A	0.60339 (11)	0.6616 (3)	0.8957 (2)	0.0530 (9)	
C10A	0.72495 (13)	0.8238 (3)	0.9324 (2)	0.0585 (9)	
N11A	0.76529 (12)	0.8350 (3)	0.9473 (2)	0.0823 (11)	
N12A	0.66396 (11)	1.0672 (3)	0.9145 (2)	0.0810 (11)	
H50A	0.6939	1.0850	0.9243	0.097*	
H40A	0.6439	1.1377	0.9092	0.097*	
C13A	0.56757 (12)	0.4028 (4)	0.9000 (3)	0.0638 (10)	
O13A	0.60412 (9)	0.3324 (2)	0.90798 (19)	0.0771 (8)	
O14A	0.52397 (8)	0.3481 (2)	0.89287 (19)	0.0786 (8)	
C15A	0.51823 (14)	0.1906 (4)	0.8995 (3)	0.0886 (14)	
H15C	0.5253	0.1586	0.9589	0.106*	
H15D	0.5396	0.1410	0.8619	0.106*	
C16A	0.46789 (17)	0.1595 (5)	0.8721 (4)	0.1174 (18)	
H16D	0.4473	0.2176	0.9056	0.176*	
H16E	0.4613	0.0583	0.8813	0.176*	
H16F	0.4623	0.1822	0.8115	0.176*	
C17A	0.67533 (11)	0.5887 (3)	0.8153 (2)	0.0547 (9)	
C18A	0.66234 (14)	0.6484 (4)	0.7353 (3)	0.0740 (11)	
H18A	0.6414	0.7270	0.7316	0.089*	
C19A	0.6800 (2)	0.5929 (6)	0.6608 (3)	0.1099 (18)	
H19A	0.6710	0.6338	0.6071	0.132*	
C20A	0.7113 (2)	0.4757 (8)	0.6657 (5)	0.128 (3)	
H20A	0.7238	0.4384	0.6156	0.154*	
C21A	0.72347 (18)	0.4168 (6)	0.7438 (5)	0.118 (2)	
H21A	0.7442	0.3378	0.7471	0.141*	
C22A	0.70605 (13)	0.4704 (4)	0.8190 (3)	0.0791 (12)	
H22A	0.7148	0.4273	0.8722	0.095*	
N1B	0.98769 (10)	0.2450 (3)	0.1030 (2)	0.0690 (9)	
N2B	0.99344 (10)	0.3898 (3)	0.1081 (2)	0.0637 (8)	
H2B	1.0203	0.4307	0.1231	0.076*	
C3B	0.95301 (11)	0.4650 (3)	0.0872 (2)	0.0517 (8)	
C4B	0.86559 (10)	0.3732 (3)	0.0388 (2)	0.0462 (8)	
H4B	0.8622	0.4317	−0.0144	0.055*	
C5B	0.84917 (11)	0.2186 (3)	0.0175 (2)	0.0490 (8)	
C6B	0.87600 (12)	0.0964 (3)	0.0311 (2)	0.0575 (9)	
O7B	0.92214 (8)	0.0970 (2)	0.06421 (18)	0.0714 (8)	
C8B	0.94166 (12)	0.2333 (3)	0.0776 (2)	0.0595 (9)	
C9B	0.91763 (11)	0.3640 (3)	0.0674 (2)	0.0478 (8)	
C10B	0.80137 (13)	0.2011 (3)	−0.0144 (2)	0.0554 (9)	
N11B	0.76248 (11)	0.1905 (3)	−0.0413 (2)	0.0784 (10)	
N12B	0.86195 (11)	−0.0413 (3)	0.0170 (2)	0.0860 (11)	
H50B	0.8332	−0.0594	−0.0027	0.103*	
H40B	0.8817	−0.1116	0.0276	0.103*	
C13B	0.95170 (12)	0.6237 (3)	0.0869 (2)	0.0547 (9)	
O13B	0.91680 (8)	0.6936 (2)	0.06285 (18)	0.0737 (8)	
O14B	0.99271 (8)	0.6791 (2)	0.11744 (18)	0.0717 (8)	
C15B	0.99710 (13)	0.8366 (3)	0.1205 (3)	0.0745 (11)	
H15A	0.9726	0.8784	0.1547	0.089*	
H15B	0.9940	0.8773	0.0623	0.089*	
C16B	1.04599 (15)	0.8670 (4)	0.1622 (3)	0.0955 (14)	
H16A	1.0480	0.8295	0.2205	0.143*	
H16B	1.0515	0.9700	0.1636	0.143*	
H16C	1.0697	0.8208	0.1292	0.143*	
C17B	0.83482 (11)	0.4393 (3)	0.1059 (2)	0.0532 (9)	
C18B	0.80440 (13)	0.5537 (4)	0.0831 (3)	0.0793 (12)	
H18B	0.8049	0.5913	0.0270	0.095*	
C19B	0.77395 (18)	0.6138 (6)	0.1384 (5)	0.1173 (19)	
H19B	0.7543	0.6912	0.1210	0.141*	
C20B	0.7730 (2)	0.5589 (8)	0.2185 (5)	0.119 (2)	
H20B	0.7518	0.5985	0.2563	0.143*	
C21B	0.8023 (2)	0.4450 (7)	0.2477 (3)	0.1114 (19)	
H21B	0.8013	0.4093	0.3041	0.134*	
C22B	0.83403 (15)	0.3849 (4)	0.1877 (3)	0.0790 (12)	
H22B	0.8542	0.3085	0.2049	0.095*	
S1	0.39614 (7)	0.6010 (2)	0.82388 (15)	0.0969 (9)	0.679 (4)
S1A	0.39306 (13)	0.6880 (5)	0.8912 (3)	0.0895 (19)	0.321 (4)
S2	0.87312 (9)	0.6108 (4)	0.8352 (3)	0.1088 (15)	0.546 (6)
S2B	0.88926 (12)	0.5896 (3)	0.7732 (3)	0.0929 (15)	0.454 (6)
O1S	0.43139 (10)	0.5574 (4)	0.9002 (3)	0.1292 (14)	
O2S	0.91562 (11)	0.5181 (3)	0.8424 (3)	0.1368 (16)	
C1S	0.40250 (19)	0.7830 (6)	0.8178 (5)	0.160 (3)	
H1S	0.4319	0.8055	0.7917	0.192*	0.679 (4)
H2S	0.4030	0.8239	0.8752	0.192*	0.679 (4)
H3S	0.3762	0.8232	0.7830	0.192*	0.679 (4)
H4S	0.4315	0.8365	0.8307	0.192*	0.321 (4)
H5S	0.3764	0.8496	0.8077	0.192*	0.321 (4)
H6S	0.4058	0.7249	0.7667	0.192*	0.321 (4)
C2S	0.34120 (18)	0.5886 (6)	0.8717 (5)	0.158 (3)	
H7S	0.3331	0.4882	0.8791	0.190*	0.679 (4)
H8S	0.3169	0.6350	0.8348	0.190*	0.679 (4)
H9S	0.3434	0.6360	0.9273	0.190*	0.679 (4)
H10S	0.3362	0.5275	0.9209	0.190*	0.321 (4)
H11S	0.3440	0.5295	0.8209	0.190*	0.321 (4)
H12S	0.3146	0.6537	0.8625	0.190*	0.321 (4)
C3S	0.8903 (2)	0.7740 (5)	0.7998 (5)	0.186 (3)	
H13S	0.9146	0.8131	0.8398	0.223*	0.546 (6)
H14S	0.8633	0.8385	0.7960	0.223*	0.546 (6)
H15S	0.9028	0.7638	0.7434	0.223*	0.546 (6)
H16S	0.9228	0.8059	0.8086	0.223*	0.454 (6)
H17S	0.8740	0.7890	0.8522	0.223*	0.454 (6)
H18S	0.8746	0.8286	0.7532	0.223*	0.454 (6)
C4S	0.8336 (3)	0.5660 (7)	0.7631 (8)	0.290 (7)	
H19S	0.8211	0.4720	0.7765	0.348*	0.546 (6)
H20S	0.8474	0.5624	0.7075	0.348*	0.546 (6)
H21S	0.8082	0.6362	0.7612	0.348*	0.546 (6)
H22S	0.8269	0.4662	0.7485	0.348*	0.454 (6)
H23S	0.8202	0.6278	0.7177	0.348*	0.454 (6)
H24S	0.8198	0.5894	0.8169	0.348*	0.454 (6)

### Atomic displacement parameters (Å^2^)

**Table:**

	*U*^11^	*U*^22^	*U*^33^	*U*^12^	*U*^13^	*U*^23^
N1A	0.0434 (18)	0.0496 (17)	0.116 (3)	0.0062 (13)	0.0079 (18)	0.0038 (17)
N2A	0.0419 (17)	0.0522 (18)	0.110 (3)	0.0025 (13)	0.0132 (17)	0.0062 (17)
C3A	0.0414 (19)	0.0483 (19)	0.079 (2)	0.0043 (16)	0.0087 (18)	0.0002 (17)
C4A	0.0442 (19)	0.0511 (18)	0.058 (2)	0.0037 (15)	0.0051 (16)	0.0021 (16)
C5A	0.0453 (19)	0.0419 (17)	0.063 (2)	0.0083 (15)	−0.0021 (16)	−0.0056 (15)
C6A	0.0456 (19)	0.049 (2)	0.076 (2)	−0.0005 (16)	−0.0011 (18)	−0.0025 (17)
O7A	0.0427 (13)	0.0423 (13)	0.116 (2)	0.0063 (10)	−0.0014 (14)	0.0028 (13)
C8A	0.046 (2)	0.048 (2)	0.091 (3)	0.0053 (16)	0.0059 (19)	0.0017 (19)
C9A	0.0428 (19)	0.0455 (18)	0.071 (2)	0.0135 (16)	0.0085 (17)	0.0045 (16)
C10A	0.052 (2)	0.0449 (19)	0.078 (3)	0.0035 (16)	−0.007 (2)	−0.0057 (17)
N11A	0.054 (2)	0.065 (2)	0.126 (3)	0.0044 (16)	−0.017 (2)	−0.0027 (19)
N12A	0.0544 (19)	0.0475 (17)	0.139 (3)	0.0040 (15)	−0.013 (2)	−0.0031 (18)
C13A	0.039 (2)	0.055 (2)	0.098 (3)	0.0024 (17)	0.013 (2)	0.001 (2)
O13A	0.0498 (15)	0.0545 (14)	0.128 (2)	0.0064 (12)	0.0092 (15)	−0.0029 (14)
O14A	0.0476 (15)	0.0529 (14)	0.135 (2)	−0.0020 (12)	0.0061 (16)	0.0037 (15)
C15A	0.068 (3)	0.050 (2)	0.148 (4)	−0.0094 (19)	0.008 (3)	0.007 (2)
C16A	0.081 (3)	0.088 (3)	0.182 (5)	−0.024 (3)	−0.006 (4)	−0.005 (3)
C17A	0.0368 (18)	0.0492 (19)	0.079 (3)	−0.0042 (15)	0.0072 (18)	−0.0056 (18)
C18A	0.074 (3)	0.070 (2)	0.079 (3)	−0.013 (2)	0.011 (2)	−0.006 (2)
C19A	0.127 (5)	0.124 (4)	0.082 (3)	−0.051 (4)	0.040 (3)	−0.025 (3)
C20A	0.095 (5)	0.142 (6)	0.153 (6)	−0.046 (4)	0.064 (5)	−0.085 (5)
C21A	0.065 (3)	0.119 (4)	0.173 (6)	0.009 (3)	0.036 (4)	−0.066 (5)
C22A	0.048 (2)	0.073 (3)	0.116 (4)	0.0119 (19)	0.010 (2)	−0.017 (2)
N1B	0.0415 (17)	0.0492 (16)	0.115 (3)	−0.0001 (13)	−0.0089 (17)	0.0045 (17)
N2B	0.0425 (17)	0.0493 (17)	0.098 (2)	−0.0002 (13)	−0.0072 (16)	0.0051 (16)
C3B	0.0394 (18)	0.0441 (18)	0.071 (2)	0.0053 (15)	0.0009 (17)	0.0052 (16)
C4B	0.0385 (17)	0.0445 (17)	0.0552 (19)	0.0047 (14)	0.0004 (15)	0.0006 (15)
C5B	0.0398 (18)	0.0418 (17)	0.064 (2)	0.0058 (14)	−0.0055 (16)	−0.0009 (15)
C6B	0.0425 (19)	0.0478 (19)	0.081 (3)	0.0004 (16)	−0.0052 (18)	0.0016 (18)
O7B	0.0514 (15)	0.0407 (13)	0.120 (2)	0.0091 (11)	−0.0164 (15)	0.0007 (13)
C8B	0.049 (2)	0.0416 (19)	0.087 (3)	0.0041 (16)	−0.0036 (19)	−0.0002 (18)
C9B	0.0418 (18)	0.0410 (17)	0.060 (2)	0.0055 (14)	−0.0018 (16)	0.0017 (15)
C10B	0.050 (2)	0.0428 (18)	0.072 (2)	−0.0001 (15)	−0.0093 (19)	−0.0079 (16)
N11B	0.056 (2)	0.0639 (19)	0.111 (3)	0.0036 (16)	−0.023 (2)	−0.0106 (18)
N12B	0.0582 (19)	0.0449 (16)	0.151 (3)	0.0027 (14)	−0.027 (2)	−0.0107 (19)
C13B	0.0408 (19)	0.052 (2)	0.071 (2)	0.0008 (16)	−0.0001 (18)	0.0024 (18)
O13B	0.0480 (15)	0.0526 (14)	0.119 (2)	0.0049 (11)	−0.0078 (15)	0.0049 (14)
O14B	0.0503 (15)	0.0462 (13)	0.117 (2)	−0.0033 (11)	−0.0111 (15)	0.0011 (13)
C15B	0.064 (3)	0.047 (2)	0.112 (3)	−0.0085 (18)	0.006 (2)	−0.006 (2)
C16B	0.083 (3)	0.076 (3)	0.126 (4)	−0.027 (2)	−0.005 (3)	−0.009 (3)
C17B	0.0435 (19)	0.0493 (19)	0.067 (2)	−0.0116 (16)	0.0032 (17)	−0.0086 (16)
C18B	0.054 (2)	0.076 (3)	0.109 (3)	0.017 (2)	0.010 (2)	−0.018 (2)
C19B	0.075 (4)	0.134 (5)	0.146 (5)	0.020 (3)	0.029 (4)	−0.053 (4)
C20B	0.082 (4)	0.135 (5)	0.145 (6)	−0.024 (4)	0.047 (4)	−0.062 (5)
C21B	0.126 (5)	0.136 (5)	0.075 (3)	−0.068 (4)	0.035 (3)	−0.029 (3)
C22B	0.087 (3)	0.077 (3)	0.074 (3)	−0.016 (2)	0.013 (2)	−0.007 (2)
S1	0.0793 (13)	0.0955 (14)	0.1160 (19)	−0.0053 (10)	0.0069 (12)	−0.0162 (12)
S1A	0.065 (2)	0.121 (4)	0.083 (3)	0.018 (2)	0.0102 (19)	0.013 (2)
S2	0.0635 (16)	0.153 (3)	0.111 (3)	0.0201 (15)	0.0120 (16)	0.056 (2)
S2B	0.0635 (19)	0.114 (2)	0.101 (3)	−0.0011 (15)	0.0044 (19)	−0.0174 (17)
O1S	0.0506 (17)	0.134 (3)	0.203 (4)	0.0091 (17)	0.006 (2)	0.077 (3)
O2S	0.067 (2)	0.092 (2)	0.245 (5)	0.0036 (17)	−0.047 (3)	0.029 (2)
C1S	0.086 (4)	0.125 (4)	0.270 (8)	0.003 (3)	0.004 (5)	0.099 (5)
C2S	0.067 (3)	0.160 (5)	0.245 (8)	−0.025 (3)	−0.012 (4)	0.086 (5)
C3S	0.127 (5)	0.088 (4)	0.333 (10)	−0.006 (3)	−0.069 (6)	0.031 (5)
C4S	0.142 (6)	0.142 (6)	0.56 (2)	0.003 (5)	−0.198 (10)	−0.020 (8)

### Geometric parameters (Å, º)

**Table:**

N1A—C8A	1.323 (4)	C13B—O14B	1.318 (4)
N1A—N2A	1.345 (4)	O14B—C15B	1.454 (4)
N2A—C3A	1.359 (4)	C15B—C16B	1.505 (5)
N2A—H2A	0.8600	C15B—H15A	0.9700
C3A—C9A	1.390 (4)	C15B—H15B	0.9700
C3A—C13A	1.450 (5)	C16B—H16A	0.9600
C4A—C9A	1.494 (4)	C16B—H16B	0.9600
C4A—C17A	1.524 (4)	C16B—H16C	0.9600
C4A—C5A	1.530 (4)	C17B—C22B	1.357 (5)
C4A—H4A	0.9800	C17B—C18B	1.387 (5)
C5A—C6A	1.356 (4)	C18B—C19B	1.358 (6)
C5A—C10A	1.407 (4)	C18B—H18B	0.9300
C6A—N12A	1.345 (4)	C19B—C20B	1.333 (8)
C6A—O7A	1.363 (4)	C19B—H19B	0.9300
O7A—C8A	1.379 (4)	C20B—C21B	1.390 (7)
C8A—C9A	1.383 (4)	C20B—H20B	0.9300
C10A—N11A	1.145 (4)	C21B—C22B	1.430 (6)
N12A—H50A	0.8600	C21B—H21B	0.9300
N12A—H40A	0.8600	C22B—H22B	0.9300
C13A—O13A	1.212 (4)	S1—O1S	1.543 (4)
C13A—O14A	1.319 (4)	S1—C1S	1.686 (5)
O14A—C15A	1.462 (4)	S1—C2S	1.749 (6)
C15A—C16A	1.476 (5)	S1—H6S	1.4746
C15A—H15C	0.9700	S1—H11S	1.6005
C15A—H15D	0.9700	S1A—C1S	1.464 (6)
C16A—H16D	0.9600	S1A—O1S	1.612 (5)
C16A—H16E	0.9600	S1A—C2S	1.728 (6)
C16A—H16F	0.9600	S2—O2S	1.463 (4)
C17A—C18A	1.378 (5)	S2—C4S	1.576 (8)
C17A—C22A	1.387 (5)	S2—C3S	1.677 (6)
C18A—C19A	1.374 (6)	S2—H17S	1.6594
C18A—H18A	0.9300	S2—H24S	1.5180
C19A—C20A	1.390 (8)	S2B—O2S	1.421 (5)
C19A—H19A	0.9300	S2B—C4S	1.573 (8)
C20A—C21A	1.344 (8)	S2B—C3S	1.744 (6)
C20A—H20A	0.9300	C1S—H1S	0.9600
C21A—C22A	1.374 (7)	C1S—H2S	0.9600
C21A—H21A	0.9300	C1S—H3S	0.9600
C22A—H22A	0.9300	C1S—H4S	0.9600
N1B—C8B	1.330 (4)	C1S—H5S	0.9600
N1B—N2B	1.344 (4)	C1S—H6S	0.9601
N2B—C3B	1.350 (4)	C2S—H7S	0.9600
N2B—H2B	0.8600	C2S—H8S	0.9600
C3B—C9B	1.379 (4)	C2S—H9S	0.9600
C3B—C13B	1.459 (4)	C2S—H10S	0.9600
C4B—C9B	1.501 (4)	C2S—H11S	0.9600
C4B—C17B	1.511 (4)	C2S—H12S	0.9600
C4B—C5B	1.524 (4)	C3S—H13S	0.9600
C4B—H4B	0.9800	C3S—H14S	0.9600
C5B—C6B	1.361 (4)	C3S—H15S	0.9600
C5B—C10B	1.408 (4)	C3S—H16S	0.9600
C6B—N12B	1.340 (4)	C3S—H17S	0.9600
C6B—O7B	1.362 (4)	C3S—H18S	0.9600
O7B—C8B	1.378 (4)	C4S—H19S	0.9600
C8B—C9B	1.382 (4)	C4S—H20S	0.9600
C10B—N11B	1.147 (4)	C4S—H21S	0.9600
N12B—H50B	0.8600	C4S—H22S	0.9600
N12B—H40B	0.8600	C4S—H23S	0.9600
C13B—O13B	1.210 (4)	C4S—H24S	0.9600
			
C8A—N1A—N2A	102.4 (3)	O1S—S1—C1S	103.6 (3)
N1A—N2A—C3A	113.3 (3)	O1S—S1—C2S	101.7 (3)
N1A—N2A—H2A	123.4	C1S—S1—C2S	100.8 (3)
C3A—N2A—H2A	123.4	O1S—S1—H6S	121.5
N2A—C3A—C9A	106.4 (3)	C2S—S1—H6S	120.0
N2A—C3A—C13A	122.4 (3)	O1S—S1—H11S	117.2
C9A—C3A—C13A	131.2 (3)	C1S—S1—H11S	120.4
C9A—C4A—C17A	113.1 (3)	H6S—S1—H11S	120.0
C9A—C4A—C5A	106.5 (2)	C1S—S1A—O1S	111.2 (4)
C17A—C4A—C5A	110.7 (3)	C1S—S1A—C2S	111.7 (4)
C9A—C4A—H4A	108.8	O1S—S1A—C2S	99.8 (3)
C17A—C4A—H4A	108.8	O2S—S2—C4S	115.8 (5)
C5A—C4A—H4A	108.8	O2S—S2—C3S	107.4 (3)
C6A—C5A—C10A	117.3 (3)	C4S—S2—C3S	102.0 (4)
C6A—C5A—C4A	125.1 (3)	O2S—S2—H17S	124.0
C10A—C5A—C4A	117.6 (3)	C4S—S2—H17S	111.9
N12A—C6A—C5A	127.1 (3)	O2S—S2—H24S	136.6
N12A—C6A—O7A	109.3 (3)	C3S—S2—H24S	110.7
C5A—C6A—O7A	123.6 (3)	H17S—S2—H24S	99.3
C6A—O7A—C8A	115.2 (2)	O2S—S2B—C4S	118.6 (5)
N1A—C8A—O7A	119.5 (3)	O2S—S2B—C3S	105.9 (3)
N1A—C8A—C9A	115.2 (3)	C4S—S2B—C3S	99.2 (4)
O7A—C8A—C9A	125.3 (3)	S1—O1S—S1A	49.26 (17)
C8A—C9A—C3A	102.8 (3)	S1A—C1S—S1	48.8 (2)
C8A—C9A—C4A	122.8 (3)	S1A—C1S—H1S	130.5
C3A—C9A—C4A	134.4 (3)	S1—C1S—H1S	109.5
N11A—C10A—C5A	178.7 (4)	S1A—C1S—H2S	61.1
C6A—N12A—H50A	120.0	S1—C1S—H2S	109.5
C6A—N12A—H40A	120.0	H1S—C1S—H2S	109.5
H50A—N12A—H40A	120.0	S1A—C1S—H3S	119.5
O13A—C13A—O14A	125.3 (3)	S1—C1S—H3S	109.5
O13A—C13A—C3A	123.5 (3)	H1S—C1S—H3S	109.5
O14A—C13A—C3A	111.2 (3)	H2S—C1S—H3S	109.5
C13A—O14A—C15A	118.5 (3)	S1A—C1S—H4S	109.6
O14A—C15A—C16A	106.2 (3)	S1—C1S—H4S	126.0
O14A—C15A—H15C	110.5	H2S—C1S—H4S	69.0
C16A—C15A—H15C	110.5	H3S—C1S—H4S	122.0
O14A—C15A—H15D	110.5	S1A—C1S—H5S	109.5
C16A—C15A—H15D	110.5	S1—C1S—H5S	124.1
H15C—C15A—H15D	108.7	H1S—C1S—H5S	117.4
C15A—C16A—H16D	109.5	H2S—C1S—H5S	82.5
C15A—C16A—H16E	109.5	H4S—C1S—H5S	109.5
H16D—C16A—H16E	109.5	S1A—C1S—H6S	109.3
C15A—C16A—H16F	109.5	S1—C1S—H6S	60.6
H16D—C16A—H16F	109.5	H1S—C1S—H6S	69.5
H16E—C16A—H16F	109.5	H2S—C1S—H6S	167.3
C18A—C17A—C22A	118.7 (4)	H3S—C1S—H6S	82.3
C18A—C17A—C4A	120.8 (3)	H4S—C1S—H6S	109.5
C22A—C17A—C4A	120.5 (3)	H5S—C1S—H6S	109.5
C19A—C18A—C17A	120.6 (4)	S1A—C2S—H7S	133.7
C19A—C18A—H18A	119.7	S1—C2S—H7S	109.5
C17A—C18A—H18A	119.7	S1A—C2S—H8S	115.3
C18A—C19A—C20A	120.0 (5)	S1—C2S—H8S	109.5
C18A—C19A—H19A	120.0	H7S—C2S—H8S	109.5
C20A—C19A—H19A	120.0	S1A—C2S—H9S	66.1
C21A—C20A—C19A	119.1 (5)	S1—C2S—H9S	109.5
C21A—C20A—H20A	120.4	H7S—C2S—H9S	109.5
C19A—C20A—H20A	120.4	H8S—C2S—H9S	109.5
C20A—C21A—C22A	121.7 (5)	S1A—C2S—H10S	109.4
C20A—C21A—H21A	119.1	S1—C2S—H10S	122.8
C22A—C21A—H21A	119.1	H7S—C2S—H10S	45.2
C21A—C22A—C17A	119.8 (5)	H8S—C2S—H10S	126.6
C21A—C22A—H22A	120.1	H9S—C2S—H10S	64.3
C17A—C22A—H22A	120.1	S1A—C2S—H11S	109.5
C8B—N1B—N2B	102.0 (3)	S1—C2S—H11S	65.0
N1B—N2B—C3B	113.4 (3)	H7S—C2S—H11S	65.4
N1B—N2B—H2B	123.3	H8S—C2S—H11S	81.9
C3B—N2B—H2B	123.3	H9S—C2S—H11S	168.7
N2B—C3B—C9B	106.8 (3)	H10S—C2S—H11S	109.5
N2B—C3B—C13B	122.2 (3)	S1A—C2S—H12S	109.5
C9B—C3B—C13B	130.9 (3)	S1—C2S—H12S	126.4
C9B—C4B—C17B	113.9 (3)	H7S—C2S—H12S	115.5
C9B—C4B—C5B	106.6 (2)	H9S—C2S—H12S	81.8
C17B—C4B—C5B	110.2 (2)	H10S—C2S—H12S	109.5
C9B—C4B—H4B	108.7	H11S—C2S—H12S	109.5
C17B—C4B—H4B	108.7	S2—C3S—H13S	109.5
C5B—C4B—H4B	108.7	S2B—C3S—H13S	121.1
C6B—C5B—C10B	117.7 (3)	S2—C3S—H14S	109.5
C6B—C5B—C4B	125.5 (3)	S2B—C3S—H14S	125.8
C10B—C5B—C4B	116.8 (2)	H13S—C3S—H14S	109.5
N12B—C6B—C5B	126.9 (3)	S2—C3S—H15S	109.5
N12B—C6B—O7B	109.1 (3)	S2B—C3S—H15S	72.2
C5B—C6B—O7B	123.9 (3)	H13S—C3S—H15S	109.5
C6B—O7B—C8B	114.8 (2)	H14S—C3S—H15S	109.5
N1B—C8B—O7B	119.1 (3)	S2—C3S—H16S	121.1
N1B—C8B—C9B	114.9 (3)	S2B—C3S—H16S	109.4
O7B—C8B—C9B	126.0 (3)	H14S—C3S—H16S	123.9
C3B—C9B—C8B	102.8 (3)	H15S—C3S—H16S	76.3
C3B—C9B—C4B	134.4 (3)	S2—C3S—H17S	72.3
C8B—C9B—C4B	122.7 (3)	S2B—C3S—H17S	109.6
N11B—C10B—C5B	178.2 (4)	H13S—C3S—H17S	76.3
C6B—N12B—H50B	120.0	H14S—C3S—H17S	63.3
C6B—N12B—H40B	120.0	H15S—C3S—H17S	172.4
H50B—N12B—H40B	120.0	H16S—C3S—H17S	109.5
O13B—C13B—O14B	125.1 (3)	S2—C3S—H18S	125.7
O13B—C13B—C3B	123.5 (3)	S2B—C3S—H18S	109.4
O14B—C13B—C3B	111.4 (3)	H13S—C3S—H18S	123.9
C13B—O14B—C15B	117.9 (3)	H14S—C3S—H18S	46.2
O14B—C15B—C16B	105.8 (3)	H15S—C3S—H18S	63.3
O14B—C15B—H15A	110.6	H16S—C3S—H18S	109.5
C16B—C15B—H15A	110.6	H17S—C3S—H18S	109.5
O14B—C15B—H15B	110.6	S2B—C4S—H19S	118.3
C16B—C15B—H15B	110.6	S2—C4S—H19S	109.5
H15A—C15B—H15B	108.7	S2B—C4S—H20S	69.1
C15B—C16B—H16A	109.5	S2—C4S—H20S	109.5
C15B—C16B—H16B	109.5	H19S—C4S—H20S	109.5
H16A—C16B—H16B	109.5	S2B—C4S—H21S	129.7
C15B—C16B—H16C	109.5	S2—C4S—H21S	109.5
H16A—C16B—H16C	109.5	H19S—C4S—H21S	109.5
H16B—C16B—H16C	109.5	H20S—C4S—H21S	109.5
C22B—C17B—C18B	118.1 (4)	S2B—C4S—H22S	109.5
C22B—C17B—C4B	121.8 (3)	S2—C4S—H22S	122.2
C18B—C17B—C4B	119.9 (3)	H20S—C4S—H22S	80.9
C19B—C18B—C17B	123.3 (5)	H21S—C4S—H22S	120.2
C19B—C18B—H18B	118.3	S2B—C4S—H23S	109.6
C17B—C18B—H18B	118.3	S2—C4S—H23S	125.9
C20B—C19B—C18B	118.3 (6)	H19S—C4S—H23S	123.9
C20B—C19B—H19B	120.9	H20S—C4S—H23S	62.2
C18B—C19B—H19B	120.9	H21S—C4S—H23S	47.3
C19B—C20B—C21B	122.7 (5)	H22S—C4S—H23S	109.5
C19B—C20B—H20B	118.6	S2B—C4S—H24S	109.4
C21B—C20B—H20B	118.6	S2—C4S—H24S	68.7
C20B—C21B—C22B	117.4 (5)	H19S—C4S—H24S	81.1
C20B—C21B—H21B	121.3	H20S—C4S—H24S	168.9
C22B—C21B—H21B	121.3	H21S—C4S—H24S	62.5
C17B—C22B—C21B	120.1 (5)	H22S—C4S—H24S	109.5
C17B—C22B—H22B	119.9	H23S—C4S—H24S	109.5
C21B—C22B—H22B	119.9		
			
C8A—N1A—N2A—C3A	−0.4 (4)	C5B—C6B—O7B—C8B	−4.7 (5)
N1A—N2A—C3A—C9A	0.6 (4)	N2B—N1B—C8B—O7B	179.4 (3)
N1A—N2A—C3A—C13A	178.7 (3)	N2B—N1B—C8B—C9B	−0.8 (4)
C9A—C4A—C5A—C6A	−12.8 (4)	C6B—O7B—C8B—N1B	−174.5 (3)
C17A—C4A—C5A—C6A	110.5 (4)	C6B—O7B—C8B—C9B	5.8 (5)
C9A—C4A—C5A—C10A	170.4 (3)	N2B—C3B—C9B—C8B	−0.9 (4)
C17A—C4A—C5A—C10A	−66.3 (4)	C13B—C3B—C9B—C8B	178.2 (3)
C10A—C5A—C6A—N12A	1.7 (5)	N2B—C3B—C9B—C4B	−179.2 (3)
C4A—C5A—C6A—N12A	−175.1 (3)	C13B—C3B—C9B—C4B	−0.1 (6)
C10A—C5A—C6A—O7A	−177.7 (3)	N1B—C8B—C9B—C3B	1.1 (4)
C4A—C5A—C6A—O7A	5.5 (5)	O7B—C8B—C9B—C3B	−179.1 (3)
N12A—C6A—O7A—C8A	−174.1 (3)	N1B—C8B—C9B—C4B	179.7 (3)
C5A—C6A—O7A—C8A	5.4 (5)	O7B—C8B—C9B—C4B	−0.6 (5)
N2A—N1A—C8A—O7A	−178.6 (3)	C17B—C4B—C9B—C3B	−65.1 (5)
N2A—N1A—C8A—C9A	0.1 (5)	C5B—C4B—C9B—C3B	173.1 (4)
C6A—O7A—C8A—N1A	171.2 (3)	C17B—C4B—C9B—C8B	116.8 (3)
C6A—O7A—C8A—C9A	−7.3 (5)	C5B—C4B—C9B—C8B	−5.0 (4)
N1A—C8A—C9A—C3A	0.2 (5)	N2B—C3B—C13B—O13B	174.8 (3)
O7A—C8A—C9A—C3A	178.8 (4)	C9B—C3B—C13B—O13B	−4.2 (6)
N1A—C8A—C9A—C4A	179.7 (3)	N2B—C3B—C13B—O14B	−6.4 (4)
O7A—C8A—C9A—C4A	−1.7 (6)	C9B—C3B—C13B—O14B	174.6 (4)
N2A—C3A—C9A—C8A	−0.5 (4)	O13B—C13B—O14B—C15B	−1.8 (5)
C13A—C3A—C9A—C8A	−178.3 (4)	C3B—C13B—O14B—C15B	179.5 (3)
N2A—C3A—C9A—C4A	−179.8 (4)	C13B—O14B—C15B—C16B	177.0 (3)
C13A—C3A—C9A—C4A	2.4 (7)	C9B—C4B—C17B—C22B	−55.6 (4)
C17A—C4A—C9A—C8A	−111.1 (4)	C5B—C4B—C17B—C22B	64.2 (4)
C5A—C4A—C9A—C8A	10.7 (4)	C9B—C4B—C17B—C18B	127.9 (3)
C17A—C4A—C9A—C3A	68.1 (5)	C5B—C4B—C17B—C18B	−112.3 (3)
C5A—C4A—C9A—C3A	−170.0 (4)	C22B—C17B—C18B—C19B	0.0 (6)
N2A—C3A—C13A—O13A	−175.5 (4)	C4B—C17B—C18B—C19B	176.5 (4)
C9A—C3A—C13A—O13A	2.1 (7)	C17B—C18B—C19B—C20B	−0.7 (7)
N2A—C3A—C13A—O14A	5.7 (5)	C18B—C19B—C20B—C21B	1.1 (9)
C9A—C3A—C13A—O14A	−176.7 (4)	C19B—C20B—C21B—C22B	−0.9 (8)
O13A—C13A—O14A—C15A	3.4 (6)	C18B—C17B—C22B—C21B	0.3 (5)
C3A—C13A—O14A—C15A	−177.8 (3)	C4B—C17B—C22B—C21B	−176.2 (3)
C13A—O14A—C15A—C16A	−169.0 (4)	C20B—C21B—C22B—C17B	0.1 (6)
C9A—C4A—C17A—C18A	54.5 (4)	C1S—S1—O1S—S1A	45.2 (3)
C5A—C4A—C17A—C18A	−64.9 (4)	C2S—S1—O1S—S1A	−59.0 (3)
C9A—C4A—C17A—C22A	−126.0 (3)	C1S—S1A—O1S—S1	−58.4 (4)
C5A—C4A—C17A—C22A	114.6 (3)	C2S—S1A—O1S—S1	59.6 (3)
C22A—C17A—C18A—C19A	−0.8 (5)	C4S—S2B—O2S—S2	−51.6 (5)
C4A—C17A—C18A—C19A	178.7 (3)	C3S—S2B—O2S—S2	58.6 (3)
C17A—C18A—C19A—C20A	−0.1 (7)	C4S—S2—O2S—S2B	49.6 (6)
C18A—C19A—C20A—C21A	0.9 (8)	C3S—S2—O2S—S2B	−63.5 (4)
C19A—C20A—C21A—C22A	−0.6 (9)	O1S—S1A—C1S—S1	51.7 (3)
C20A—C21A—C22A—C17A	−0.4 (8)	C2S—S1A—C1S—S1	−58.8 (4)
C18A—C17A—C22A—C21A	1.1 (6)	O1S—S1—C1S—S1A	−51.9 (3)
C4A—C17A—C22A—C21A	−178.4 (4)	C2S—S1—C1S—S1A	53.0 (3)
C8B—N1B—N2B—C3B	0.2 (4)	C1S—S1A—C2S—S1	62.2 (4)
N1B—N2B—C3B—C9B	0.5 (4)	O1S—S1A—C2S—S1	−55.3 (3)
N1B—N2B—C3B—C13B	−178.7 (3)	O1S—S1—C2S—S1A	59.9 (3)
C9B—C4B—C5B—C6B	6.1 (4)	C1S—S1—C2S—S1A	−46.6 (3)
C17B—C4B—C5B—C6B	−118.0 (4)	O2S—S2—C3S—S2B	57.7 (3)
C9B—C4B—C5B—C10B	−177.6 (3)	C4S—S2—C3S—S2B	−64.5 (5)
C17B—C4B—C5B—C10B	58.3 (4)	O2S—S2B—C3S—S2	−59.7 (3)
C10B—C5B—C6B—N12B	0.3 (6)	C4S—S2B—C3S—S2	63.7 (5)
C4B—C5B—C6B—N12B	176.6 (3)	O2S—S2B—C4S—S2	51.5 (4)
C10B—C5B—C6B—O7B	−177.9 (3)	C3S—S2B—C4S—S2	−62.3 (4)
C4B—C5B—C6B—O7B	−1.5 (5)	O2S—S2—C4S—S2B	−47.8 (5)
N12B—C6B—O7B—C8B	176.9 (3)	C3S—S2—C4S—S2B	68.4 (4)

### Hydrogen-bond geometry (Å, º)

Cg is the centroid of the N1B–C9B/C8B ring.

**Table:**

*D*—H···*A*	*D*—H	H···*A*	*D*···*A*	*D*—H···*A*
N2*A*—H2*A*···O1*S*	0.86	1.90	2.737 (4)	165
N2*B*—H2*B*···O2*S*^i^	0.86	1.90	2.750 (5)	168
N12*A*—H50*A*···N11*B*^ii^	0.86	2.19	3.024 (5)	164
N12*A*—H40*A*···O13*A*^iii^	0.86	2.11	2.958 (4)	170
N12*B*—H50*B*···N11*A*^iv^	0.86	2.23	3.072 (5)	165
N12*B*—H40*B*···O13*B*^v^	0.86	2.10	2.945 (4)	168

Symmetry codes: (i) −*x*+2, −*y*+1, −*z*+1; (ii) *x*, *y*+1, *z*+1; (iii) *x*, *y*+1, *z*; (iv) *x*, *y*−1, *z*−1; (v) *x*, *y*−1, *z*.
